# Strong epistatic and additive effects of linked candidate SNPs for *Drosophila* pigmentation have implications for analysis of genome-wide association studies results

**DOI:** 10.1186/s13059-017-1262-7

**Published:** 2017-07-03

**Authors:** Jean-Michel Gibert, Jorge Blanco, Marlies Dolezal, Viola Nolte, Frédérique Peronnet, Christian Schlötterer

**Affiliations:** 1 Institut für Populationsgenetik, Vetmeduni Vienna, Veterinärplatz 1, 1210 Wien, Austria; 2 Sorbonne Universités, UPMC Univ Paris 06, CNRS, Biologie du Développement Paris Seine-Institut de Biologie Paris Seine (LBD-IBPS), case 24, 9 quai St-Bernard, 75005 Paris, France

**Keywords:** Genome-wide associations studies (GWAS), Genetic architecture, Additivity, Epistasis, Pigmentation, Drosophila

## Abstract

**Background:**

The mapping resolution of genome-wide association studies (GWAS) is limited by historic recombination events and effects are often assigned to haplotype blocks rather than individual SNPs. It is not clear how many of the SNPs in the block, and which ones, are causative. *Drosophila* pigmentation is a powerful model to dissect the genetic basis of intra-specific and inter-specific phenotypic variation. Three tightly linked SNPs in the t-MSE enhancer have been identified in three *D. melanogaster* populations as major contributors to female abdominal pigmentation. This enhancer controls the expression of the pigmentation gene *tan (t)* in the abdominal epidermis. Two of the three SNPs were confirmed in an independent study using the *D. melanogaster* Genetic Reference Panel established from a North American population.

**Results:**

We determined the functional impact of SNP1, SNP2, and SNP3 using transgenic lines to test all possible haplotypes in vivo. We show that all three candidate SNPs contribute to female *Drosophila* abdominal pigmentation. Interestingly, only two SNPs agree with the effect predicted by GWAS; the third one goes in the opposite direction because of linkage disequilibrium between multiple functional SNPs. Our experimental design uncovered strong additive effects for the three SNPs, but we also found significant epistatic effects explaining up to 11% of the total variation.

**Conclusions:**

Our results suggest that linked causal variants are important for the interpretation of GWAS and functional validation is needed to understand the genetic architecture of traits.

**Electronic supplementary material:**

The online version of this article (doi:10.1186/s13059-017-1262-7) contains supplementary material, which is available to authorized users.

## Background

Over the past years, genome-wide association studies (GWAS) have become the method of choice to identify functionally relevant variation for a wide set of traits [1, 2]. Despite the increased resolution of GWAS, compared with experimental crosses, the mapping resolution is frequently not sufficiently high to map the causative variants. The haplotype blocks identified by GWAS often carry multiple variants and it is not clear how many of them and which ones are causative. Many approaches have been proposed to prioritize candidate single nucleotide polymorphisms (SNPs). While rather advanced approaches are available for protein-coding variants (e.g. SIFT, PolyPhen-2, MutationAssessor [3–5]), the characterization of regulatory SNPs suffers from low sequence conservation and incomplete knowledge about functionally important motives. The large number of candidate SNPs also precludes experimental inference of epistasis since the number of possible haplotypes scales exponentially with the number of loci. Indeed, only a few studies analyzed the fitness consequences of all combinations of small sets of mutations (reviewed in [6]). Consequently, the experimental validation of GWAS results is often confined to cell culture or knockdown of candidate genes.

*Drosophila* pigmentation has a long tradition as model to study the genetic basis of phenotypic variation within and between species [7]. Pool-GWAS in three natural *D. melanogaster* populations identified three closely linked SNPs as major contributors to female abdominal pigmentation [8, 9]. These three SNPs are located within a 208-bp window of the *t-MSE* enhancer, which controls the expression of the pigmentation gene *tan (t)* in the abdominal epidermis [10]: X-9121129 (SNP1), X-9121094 (SNP2), and X-9120922 (SNP3) (Additional files 1 and 2: Table S1 and Figure S1). An independent study, which does not rely on Pool-Seq [11] of extreme phenotypes but analyzed individual isofemale lines, confirmed the importance of this regulatory region for natural variation in female abdominal pigmentation [12]. Using the *D. melanogaster* Genetic Reference Panel, which was established from a North American population, two of the SNPs identified by Pool-GWAS (SNP1, SNP2) were also detected [12].

Building on the previously identified GWAS signal in the regulatory region of the pigmentation gene *tan (t)*, we performed the first in vivo characterization of all GWAS candidate SNPs in a quantitative trait locus (QTL) region. It could have been expected that a single causative variant was linked to two neutral SNPs. However, we find that all three candidate SNPs have strong additive and complex epistatic effects on pigmentation. Interestingly, only two agree with the effect predicted by GWAS; the third one goes in the opposite direction.

## Results

**Fig. 1:**
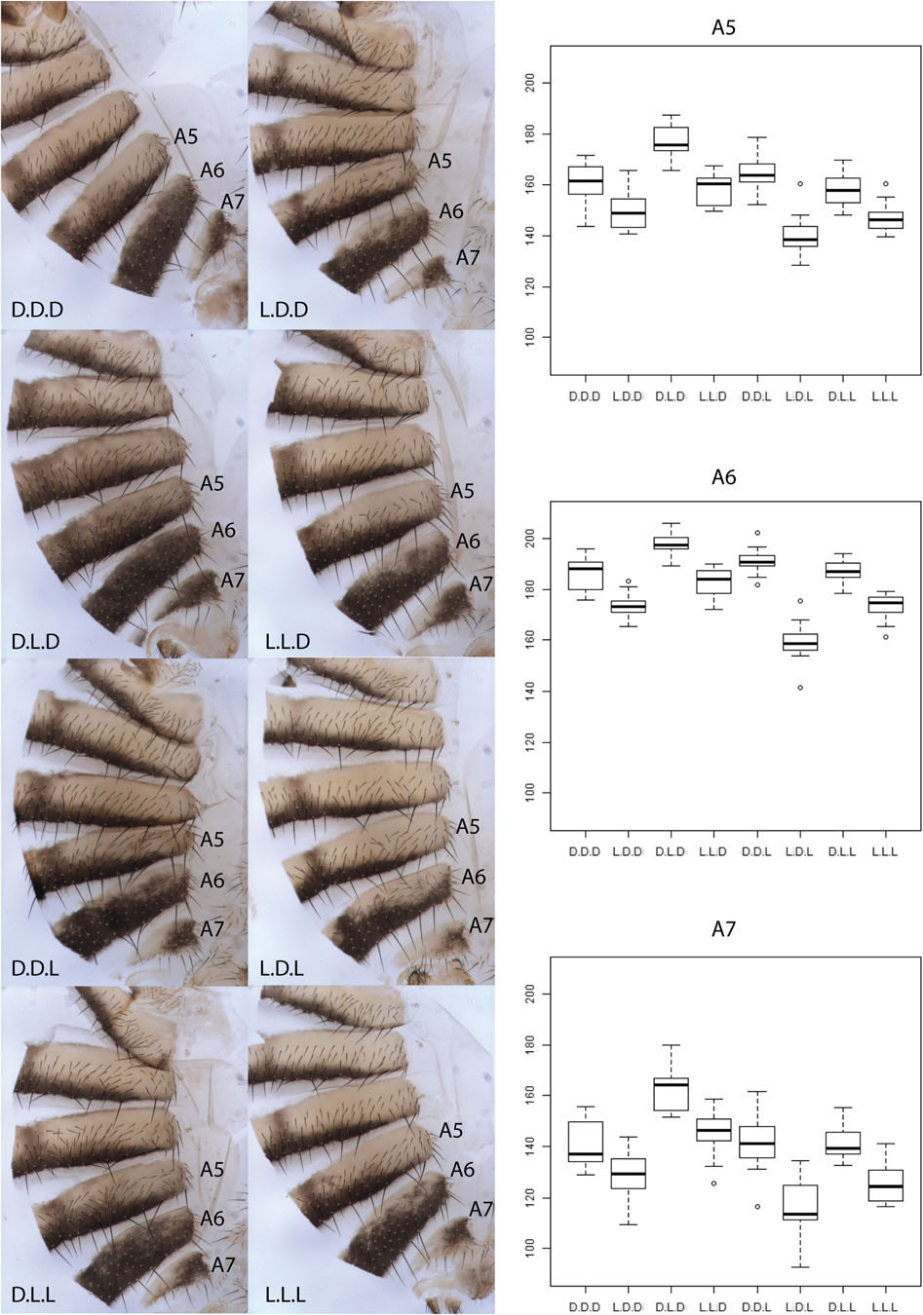
Effect of the three SNPs on abdominal pigmentation. *Left*: abdominal cuticles of the different genotypes carrying the eight possible combinations of the three tested SNPs (1, 2, and 3). A5, A6, and A7: abdominal segments 5, 6, and 7. *Right*: *box plots* of pigmentation of A5, A6, and A7 for the eight genotypes carrying all possible combinations of the three tested SNPs. The letters D and L correspond to the prediction of the GWAS studies (*Dark* or *Light*)

We used transgenic lines to evaluate the functional impact of SNP1, SNP2, and SNP3. We constructed rescue transgenes, each containing the *t-MSE* with one of the eight combinations of the three SNPs fused to the *hsp70* minimal promoter and the *t* complementary DNA (cDNA). Each transgene was inserted at the same genomic position using phiC31 integrase-based transgenesis [13]. The effect of the different SNP combinations on abdominal pigmentation was assessed in females with an otherwise identical genetic background, mutant for *t* (allele *t* ^*d07784*^ [14]). The different genotypes showed pronounced pigmentation differences (Fig. 1, left). For a quantitative analysis of pigmentation differences, we analyzed 15 individuals for each genotype and quantified the pigmentation in segments A5, A6, and A7 on mounted abdominal segments (Additional file 3: Raw Data; Fig. 1, right). We detected clear differences between the genotypes, covering a similar phenotypic space as wild-type (WT) flies (Fig. 1). We determined the main effects of all SNPs and their interactions using ANOVA (Additional file 4: Table S2). All SNPs had very significant main effects in all segments (*p* < 0.001), explaining up to 71% of the total variance in pigmentation (Table 1). SNP1 had the strongest main effect, explaining 29–53% of the variation. Epistatic effects were considerably weaker accounting for 6–11% of the total variation. For all segments, a model with epistatic effects provided a significantly better fit to the data than a model with main effects only (*p* ≤ 0.001). The presence of epistatic effects in a regulatory element is in line with results for transcription factor binding sites [15].

**Table 1:** Summary of the three-way ANOVAs (full factorial model) performed independently on segments A5, A6, and A7

	A5		A6		A7	
	*p* value	Eta Squared	*p* value	Eta Squared	*p* value	Eta Squared
1	<0.001	0.422	<0.001	0.533	<0.001	0.295
2	<0.001	0.059	<0.001	0.097	<0.001	0.132
3	<0.001	0.130	<0.001	0.081	<0.001	0.180
1 × 2	0.206	0.004	<0.001	0.025	0.394	0.002
1 × 3	0.260	0.003	<0.001	0.032	0.074	0.009
2 × 3	<0.001	0.058	0.013	0.010	<0.001	0.047
1 × 2 × 3	<0.001	0.044	<0.001	0.042	0.025	0.015

1, 2, and 3 in the first column correspond to SNP1, SNP2, and SNP3. *P* values for the different effects are indicated as well as Eta Squared, a measure of the effect size

The effects of the three SNPs on the pigmentation were largely consistent across segments. Nevertheless, some differences between segments were noticed (Fig. 1, Table 1, Additional file 4: Table S2). SNP1 had the strongest main effect in A6 and the weakest main effect in A7, whereas the opposite was observed for SNP3. SNP2 had the strongest main effect in A7 and the weakest main effect in A5. The two-way interactions involving SNP1 were only significant in A6, while all other interactions were significant across all three segments (Table 1). We attribute this heterogeneity among segments to differences in expression of genes regulating *tan*. The transcription factors Abdominal-B and bric-à-brac (BAB1 and BAB2), which regulate abdominal pigmentation and are expressed at an increasing level along the anteroposterior axis in pupal epidermis [16, 17], are good candidates, although the polymorphic SNPs are not located in characterized binding sites.

Importantly, not all *Dark* alleles predicted by GWAS increased pigmentation in our functional assays. While the *Dark* alleles of SNP1 and SNP3 increase pigmentation, the *Dark* allele of SNP2 has the opposite effect. Indeed, the darkest genotype corresponds to the combination D.L.D, whereas the lightest genotype corresponds to the opposite combination L.D.L. Thus, opposite to the predictions of three GWAS studies, SNP2 does not affect pigmentation in the same direction as the linked SNPs. A closer inspection of the underlying haplotype structure shows that despite the high recombination rate and large population size of *D. melanogaster*, the absence of the haplotypes with extreme pigmentation prevented the correct inference of the effects of the three causative SNPs [9].

## Discussion

The functional impact of natural variants on a given trait is one of the most pressing questions in genetics. GWAS and QTL mapping studies substantially advanced our understanding of the contribution of genes at different, well-separated locations in the genome. Nevertheless, the analysis of linked variants has been severely limited by linkage disequilibrium. In this report, we use, to our knowledge for the first time, transgenesis to break up linkage disequilibrium among closely linked sites to determine their contribution to the trait of interest (pigmentation) and to study the underlying genetic architecture. Our result of all candidate SNPs of the *tan* locus contributing to the pigmentation demonstrates that multiple SNPs could be contributing to a single QTL. The current literature already indicates that this phenomenon may be not uncommon: several multiparent advanced intercrosses noted that a given QTL explained a significant fraction of the variation, but variants segregating in the corresponding region did not [18–21]. The authors interpreted this discrepancy as evidence for multiple functional alleles contributing to the trait [18–20]. Similarly, multiple alleles have been suggested to contribute to a given eQTL [22, 23]. The strongest independent support for multiple functional variants comes from a study on the *Alcohol dehydrogenase* (*Adh*) in *D. melanogaster*, where the authors divided the gene into three parts with multiple SNPs and showed that each part contributed to *Adh* expression [24].

Analyzing all eight possible combinations of the three adjacent functional intra-specific variants provided the first *in vivo* analysis of the genetic architecture of a regulatory module in a higher eukaryote. Although additive effects explain most of the trait variation, up to 11% of the variation arises from epistatic interactions between the three sites. The regulatory architecture of pigmentation is similar in the three abdominal segments, but some noticeable differences are present. The about tenfold difference in the variance, explained by epistatistic two-way interactions involving SNP1, results in a significant effect only in A6. In addition to cuticle pigmentation, *tan* is also involved in vision [14]. The expression of *tan* in photoreceptors is responsible for the hydrolysis of carcinine, a different substrate than Beta-alanyl-dopamine which is responsible for cuticle pigmentation [25, 26]. We anticipate that such other roles of *tan* could have their own regulatory architecture, which differs from the one of abdominal pigmentation.

Our observations have direct implications for other GWAS studies: (1) consistent with a previous report concluding that complex traits are mainly governed by additive effects [27], we find that the main effects are much stronger than epistasis; (2) linkage disequilibrium between causal SNPs could result in predicting effects in the wrong direction. Very importantly, this is not an artifact limited to this specific case in *Drosophila*, it will apply to all GWAS studies [28]. The confounding effect of multiple causative alleles at the same locus has also been described for several other systems (e.g. [28, 29]).

## Conclusion

We propose that the possibility of linked causal variation needs to receive more attention and emphasize the importance of experimental systems, such as fruit flies, which allow functional testing of candidate SNPs *in vivo* as well as the inference of the underlying genetic architecture.

## Methods

### Determination of the ancestral state

In order to determine the ancestral state of the SNPs, the *t_MSE* of *D. melanogaster* was aligned with those of *D. simulans, D. sechellia*, and *D. yakuba* using clustalw (http://www.genome.jp/tools/clustalw/). These sequences were obtained by blasting the *t_MSE* of *D. melanogaster* against the genomes of these species (https://blast.ncbi.nlm.nih.gov/Blast.cgi).

### Drosophila strains and culture

Flies were reared on standard medium at 25 °C. *y* ^*1*^ *w* ^*1118*^ flies were used as a source of genomic DNA. Targeted transgenesis was carried out using the line *ZH-attP-86Fb* (BL-24749), which harbors the *attP* landing site at the cytological location 86 F8. The *tan* mutant allele *t* ^*d07784*^ was obtained from the Bloomington Drosophila Stock center (BL-19282).

### Reporter constructs and targeted transgenesis

The *MSE* element was amplified by PCR using genomic DNA from *y* ^*1*^ *w* ^*1118*^ flies (with primers *MSE0-F* and *MSE0-R* listed below) and cloned into the pGEMT-Easy vector (Promega). The resulting plasmid, *pGEMT-MSE*, was used as a template to generate *MSE* elements containing the different SNPs combinations following the site-directed plasmid mutagenesis technique (QuickChange™, [30]). The *MSE* element from the *y* ^*1*^ *w* ^*1118*^ flies carries the nucleotides predicted to give a dark pigmentation for each of the three SNPs [8]. The primer pairs *MSE1-F* and *MSE1R*, *MSE2-F* and *MSE2-R*, and *MSE3-F* and *MSE3-R* (listed below) were used to mutate them into the nucleotides predicted to give a light pigmentation. A reporter construct, generated using restriction-enzyme-based strategies, was used to analyze *MSE* enhancer activity. This vector derives from the site-specific transformation vector *pattB* (kindly provided by Johannes Bischof) and carries the *tan* cDNA (*RH41996*, *DGRC# 17763*) as reporter gene. The different *MSE* elements were cloned upstream of the *Hsp-70* minimal promoter and the *tan* cDNA. The resulting constructs were inserted at the same genomic location on the third chromosome via site-specific integration [13]. Such mini-genes where an enhancer is cloned just upstream the cDNA of the gene it drives have been previously fruitfully used as sensitive and quantitative read-out of enhancer function [31, 32].

### Establishment of the stocks for the phenotyping

We established homozygous transgenic stocks by crossing the transgenic flies with balancer stocks. In order to homogenize the genetic background, the transgenic flies were backcrossed for ten generations to the stock *ZH-attP-86Fb* (BL-24749), which had been used for injections. The transgenes were followed by the red eye color from the functional *w* locus of the vector. The X-chromosome carrying a transgene with the phiC31 integrase was later replaced with an X-chromosome carrying the *tan* mutant allele (*w* ^*1118*^ *t* ^*d0778*4^ [14]), whereas the autosomes were maintained using balancer chromosomes. We checked by Sanger sequencing that the lines carried the right transgenes. We observed that flies mutant for *tan* and homozygous for the rescue transgenes had a very pronounced pigmentation, much darker than WT at 25 °C. We therefore phenotyped flies heterozygous for the transgene and homozygous for the *t* ^*d07784*^ allele. These flies were obtained by crossing transgenic flies homozygous for the transgenes and for the *t* ^*d07784*^ allele with the *t* ^*d07784*^ stock.

### Abdominal cuticle preparations

Four-day-old flies were stored for eight days in 75% ethanol. Abdomens were cut next to the dorsal midline and the abdominal cuticles were cleaned, dehydrated in 100% ethanol, and mounted in Euparal (Roth). Fifteen female flies were processed for each genotype condition.

### Imaging of fly abdomens

Cuticle preparations were imaged with a binocular equipped with Leica DC480 digital camera using the *Leica IM50 Image Manager* software. An annular lamp was used to ensure homogeneous lighting. All pictures were taken in the same session and identical settings were used throughout the session. Pigmentation was quantified in A5, A6, and A7 hemisegments as the mean intensity using ImageJ software as previously reported [33]. For this, the hemisegments were delimited by hand and the function “measure” of ImageJ used. The obtained values were subtracted from 255 to get values comprised between 0 (white) and 255 (black).

### Statistical analyses

All statistical analyses were performed with R 3.2.3 [34, 35]. A full factorial three-way ANOVA was performed with sum zero constraints (option *contr.sum*) and estimation of Type III SS with package “*car*.” Effect sizes were estimated as Eta Squared values, calculated as SS_effect_/SS_Total_.

### List of primers used in the study

- *MSE0-F* 5′ GCGTTCCAACACCCCGTCTAATCTA 3′
- *MSE0-R* 5′ CACGATTTCCGTATTTGAAATAATA 3′
- *MSE1-F* 5′ ATAATATATTTATATTCTGATTATT 3′
- *MSE1-R* 5′ AATAATCAGAATATAAATATATTAT 3′
- *MSE2-F* 5′ AATTATCCTAAGCCTTGATTCTATC 3′
- *MSE2-R* 5′ GATAGAATCAAGGCTTAGGATAATT 3′
- *MSE3-F* 5′ TCTAATTAGTATACATATTATGATC 3′
- *MSE3-R* 5′ GATCATAATATGTATACTAATTAGA 3′

## Additional files

Additional file 1: Table S1. Information related to the three most significant SNPs involved in female abdominal pigmentation variation in segment A7 identified previously [8]. (DOCX 50 kb)

Additional file 2: Figure S1 Alignment of *t_MSE* from *D. melanogaster*, *D. simulans*, *D. sechellia*, and *D. yakuba*. (DOCX 108 kb)

Additional file 3: Pigmentation measures in segments A5, A6, and A7 for the eight genotypes analyzed in this study. 1 and 2 in the SNP columns correspond to D or L alleles, respectively. (XLSX 43 kb)

Additional file 4: Table S2. ANOVAs performed independently on segments A5, A6, and A7 to test the effect of the three SNPs on pigmentation. (DOCX 101 kb)
