# Supplementary material for: Strong epistatic and additive effects of linked candidate SNPs for Drosophila pigmentation have implications for analysis of genome-wide association studies results
Source: Genome Biol. 2017 Jul 3;18:126. doi: 10.1186/s13059-017-1262-7 (PMC5496195; doi:10.1186/s13059-017-1262-7)
Supplement: Supplementary file 1 — Information related to the three most significant SNPs involved in female abdominal pigmentation variation in segment A7 identified previously [8]. (DOCX 50 kb) [file 13059_2017_1262_MOESM1_ESM.docx]

**Additional File 1 Table S1:** Information related to the three most significant SNPs involved in female abdominal pigmentation variation in segment A7 identified previously [8]. Ancestral states of the SNPs were determined using the alignment of *Drosophila melanogaster t_MSE* with those of other species (see Additional File 2: Figure S1).

| Rank | 1 | 2 | 3 |
| --- | --- | --- | --- |
| p-value | 1.39E-58 | 2.06E-56 | 6.84E-51 |
| Position | X-9121129 | X-9121094 | X-9120922 |
| Allele in Dark flies | C | T | G |
| Allele in Light flies | T | C | A |
| Ancestral state | C | C | A |
