# Supplementary material for: Strong epistatic and additive effects of linked candidate SNPs for Drosophila pigmentation have implications for analysis of genome-wide association studies results
Source: Genome Biol. 2017 Jul 3;18:126. doi: 10.1186/s13059-017-1262-7 (PMC5496195; doi:10.1186/s13059-017-1262-7)
Supplement: Supplementary file 2 — Alignment of t_MSE from D. melanogaster, D. simulans, D. sechellia, and D. yakuba. (DOCX 108 kb) [file 13059_2017_1262_MOESM2_ESM.docx]

**Additional File 2 Figure S1:** Alignment of *t_MSE* from *D. melanogaster, D. simulans, D. sechellia* and *D. yakuba*. The three SNPs are indicated in color in the *D. melanogaster* sequence.

sim GTGTCCCAACACCCCGTCTAATCTAAGATCTATTTATTGCA--TTTTTTTTCGTAGCTTC

sec GTGTCCCAACACCCCGTCTAATCTAAGATCTATTTATTAC---TTTTTTTTCGTAGCTTC

mel GCGTTCCAACACCCCGTCTAATCTAAGATCTATTTATTGCACTTTTTTTTTCCTAGCTTC

yak GTGTACCAACAGCCCGCCTAATCTAAGATCTATTTATTGCA--TTTTTTTTCGTAGCTTC

* ** ****** **** ********************* * ********* *******

sim GTAAGTCGA--TAGTATCAGTTTTAAGATTTAAGCGATAAATAAAGAAATGTTTGTAAAC

sec GTAAGTCGA--TAGTATCAGTTTTAAGATTTAAGCGATAAATAAAGAAATGTTTGTAAAC

mel GTAAGTCGA--TAG--TCAGTTTTCAGATTTAAGCGATAAATAAATAAATGTTTGTAAAC

yak GTAAGTTGTATTAGTATCAGTTTTAAGATTTAAGCGATAAATAAAGAAATGTTAATAGAC

****** * *** ******** ******************** ******* ** **

sim ACTCAAAGAAATGTTATTCGATTTCTAAGAATCAAGTTGTTAGC----------------

sec ACTCAAAGAAATGT----------------------------------------------

mel ACTTAAAGAAATGTTATTCAATTTCTAGGAATCAAGTTGTTAGAAGTAATAAACAAAAGG

yak ACTGAAAGAAATGTTATTACACTTTTCAGAAAATAATTGACACACGT-------------

*** **********

sim TAAACTTATTTGTT-TAT--GTCTAT--GTCTCAACACAATCCTAGC-AGTTGGAAAGCA

sec -AAACTTATTTGCT-TAT--GTTTAT--GTCTCAACGCAATCCTATCCAGTTGGAAAGCA

mel TTAACTTATTTGTT-TATTTATTTATTTGTTTCAACTCAATCCTAGC-AGTTGGAAAGCA

yak CAAACTCCTTTGAAATATCAATTAAA---TGTTCATGTTTTCTCAGT---TTATAGTGCA

**** **** *** * * * * * ** * ** * ***

sim CCAAGACCACTTGAAAATTATAAGGTAATAAAA-CGCAAATCTGAATAGTTGAAGTATTA

sec CCAAGACCACTTGAAAATTATAAAGTAATAAAAACGCAACTCTGAATAGTTGAAGTATTA

mel CCAAGACCACTTGAAAATTATAATGTAATAAAAACGCAAATTTGAATAGTTGAAGTAATA

yak CAAACACCACTTGGAAAA-ACCACTTATCGAGTAATAAAA--AAAATAGTTGCGGTAATA

* ** ******** *** * * ** * ** ******** *** **

sim AAAAAAA-GAGACTGTCTAATTAGTATACAAATTATGATCTTGAATTCGCAAATTAGTTT

sec AAAAAAAAGAGACTGTCTAATTAGTATACAAATTATGATCTTGAATTCGCAAATTAGTTT

mel AAAAAAA-GAGACTGTCTAATTAGTATGCATATTATGATCTTGAATTCTCAAATTAGTTT

yak AAAAAGA---GACTGTCCAATGAATATACAAATTATGATCTTGAATTCGCAAATTGGTTG

***** * ******* *** * *** ** ***************** ****** ***

sim ATTTAAACACTAAAATCTAATATTCTAATGGTGCAAGAGTAAAATGCACTCATTTTTGTT

sec ATTTAAACACTAAAATCTAATATTCTAATGGTGCAAGAGTAAAATGCACTCATTTTTGTT

mel ATTTAAACACTAAAATCTAATATTCTAATGGTGCAAGAGTAAAATGCACTCATTTTTGTT

yak ATTTAAGCACTAAAAATAAATATTCTAATGGTCCAGGAGTGAAATGCACCTATTTTTGTT

****** ******** ************** ** **** ******** *********

sim ATTGACTTCAACTTTGGATAATA-CCGGTTCACTTGGCAATGGGAACTTAAGTATACTAC

sec ATTGACTTCAACTTTGGATAATA-CCGGTTTACTTGGCAATGGGAACTTA----TACTAC

mel ATTGACTTCAACTTTGGATACCAACCGGTTCACTTGGCAATGGGAATTTAAGTATACTAC

yak ATTGATTTCAACTTTAGATA----CCGGTTCACTCAGCCATGGGAATTTAAGTATGCTAC

***** ********* **** ****** *** ** ******* *** * ****

sim TTAATGCAATTATCCTAAGCCTTGATTATAT----------------CTATAAAACGTAT

sec TTAATGCAATTATCCTAAGCCTTGATTATAT----------------TTATAAAACGTAT

mel TTAGTGCAATTATCCTAAGTCTTGATTCTAT----------------CTATAAAACGTAT

yak TAAATGCAATTATCCTAAGCCTTCATTCTACTTATTAAACTATAAAACTATAAAACGTAG

* * *************** *** *** ** ***********

sim AACATATTTACAAGCATTCTTTAGTTTCTAATTGTTTAT-TATTATTT-TAAATACGGAA

sec AACATATTTACAAGCATT--------TCTAATTGTTTAT-TATTATTT-TAAATACGGAA

mel AATATATTTACA-------------TTCTGATTATTTAT-TATTATTT-CAAATACGGAA

yak ACTGTAGGTACAATCAATCTGTAGTTTCTGGTTATTTATATACTACTTATAAATACGGAA

* ** **** *** ** ***** ** ** ** **********

sim ATCGAG

sec ATCGAG

mel ATCGTG

yak ATCGAG

**** *

SNP1 C

SNP2 T

SNP3 G
