# Supplementary material for: Strong epistatic and additive effects of linked candidate SNPs for Drosophila pigmentation have implications for analysis of genome-wide association studies results
Source: Genome Biol. 2017 Jul 3;18:126. doi: 10.1186/s13059-017-1262-7 (PMC5496195; doi:10.1186/s13059-017-1262-7)
Supplement: Supplementary file 4 — ANOVAs performed independently on segments A5, A6, and A7 to test the effect of the three SNPs on pigmentation. (DOCX 101 kb) [file 13059_2017_1262_MOESM4_ESM.docx]

**Additional File 4 Table S2:** ANOVAs performed independently on segments A5, A6 and A7 to test the effect of the three SNPs on pigmentation. Full factorial models were used.

Effects of the three SNPs on pigmentation in A5

|  | D.F. | SS | MS | F | Prob>F |
| --- | --- | --- | --- | --- | --- |
| 1 | 1 | 8,068.751 | 8,068.751 | 168.846 | <0.001 |
| 2 | 1 | 1,129.079 | 1,129.079 | 23.627 | <0.001 |
| 3 | 1 | 2,492.178 | 2,492.178 | 52.151 | <0.001 |
| 1x2 | 1 | 77.237 | 77.237 | 1.616 | 0.206 |
| 1x3 | 1 | 61.366 | 61.366 | 1.284 | 0.260 |
| 2x3 | 1 | 1,101.581 | 1,101.581 | 23.052 | <0.001 |
| 1x2x3 | 1 | 852.144 | 852.144 | 17.832 | <0.001 |
| Error | 112 | 5,352.206 | 47.788 |  |  |
| Total | 119 | 19,134.542 | 160.794 |  |  |

Effects of the three SNPs on pigmentation in A6

|  | D.F. | SS | MS | F | Prob>F |
| --- | --- | --- | --- | --- | --- |
| 1 | 1 | 10,245.829 | 10,245.829 | 333.113 | <0.001 |
| 2 | 1 | 1,856.439 | 1,856.439 | 60.357 | <0.001 |
| 3 | 1 | 1,557.548 | 1,557.548 | 50.639 | <0.001 |
| 1x2 | 1 | 480.472 | 480.472 | 15.621 | <0.001 |
| 1x3 | 1 | 623.936 | 623.936 | 20.285 | <0.001 |
| 2x3 | 1 | 195.892 | 195.892 | 6.369 | 0.013 |
| 1x2x3 | 1 | 811.002 | 811.002 | 26.367 | <0.001 |
| Error | 112 | 3,444.878 | 30,758 |  |  |
| Total | 119 | 19,215.996 | 161,479 |  |  |

Effects of the three SNPs on pigmentation in A7

|  | D.F. | SS | MS | F | Prob>F |
| --- | --- | --- | --- | --- | --- |
| 1 | 1 | 9,003.214 | 9,003.214 | 103.212 | <0.001 |
| 2 | 1 | 4,046.782 | 4,046.782 | 46.392 | <0.001 |
| 3 | 1 | 5,491.015 | 5,491.015 | 62.948 | <0.001 |
| 1x2 | 1 | 63.744 | 63.744 | 0.731 | 0.394 |
| 1x3 | 1 | 284.420 | 284.420 | 3.261 | 0.074 |
| 2x3 | 1 | 1,449.075 | 1,449.075 | 16.612 | <0.001 |
| 1x2x3 | 1 | 451.368 | 451.368 | 5.174 | 0.025 |
| Error | 112 | 9,769.799 | 87.230 |  |  |
| Total | 119 | 30,559.416 | 256.802 |  |  |
